# Retrospective analysis of thyroid functions and sonographic characteristics of the thyroid gland in a health check-up population in China during 2020–2024

**DOI:** 10.3389/fpubh.2026.1838770

**Published:** 2026-09-17

**Authors:** Yicheng Wang, Yue Xi, Huijie Wang, Yihang Xu, Zhijun Hong

**Affiliations:** 1 The Health Management Center, The First Affiliated Hospital of Dalian Medical University, Dalian, Liaoning, China; 2 College of Basic Medical Sciences, Dalian Medical University, Dalian, Liaoning, China; 3 School of Public Health, Dalian Medical University, Dalian, Liaoning, China

**Keywords:** cross-sectional study, health check-up, thyroid function, TSH-first strategy, ultrasonography

## Abstract

**Objectives:**

This study aimed to investigate the epidemiological characteristics and temporal trends of thyroid structural and functional abnormalities among adults undergoing health examinations in Dalian, a coastal city where iodine intake levels raise concerns.

**Methods:**

This single-center retrospective cross-sectional study included adults who underwent thyroid ultrasonography and function testing at the Health Management Center of the First Affiliated Hospital of Dalian Medical University from January 2020 to December 2024. Prevalence and temporal trends were analyzed according to sex, age, and year.

**Results:**

The overall prevalence of thyroid ultrasound abnormalities was 74.85%, including nodules (49.29%), cysts (36.91%), heterogeneous echotexture (22.96%), and suspected parathyroid lesions (2.91%). Thyroid function abnormalities were detected in 7.57% of participants, predominantly driven by TSH alterations (86.32% of functional disorders). Thyroid autoantibody positivity reached 33.02%. From 2020 to 2024, structural abnormalities showed a gradual upward trend, whereas thyroid function and antibody abnormalities declined. Females had slightly higher rates of structural abnormalities and markedly higher rates of thyroid function abnormalities (9.05% vs. 5.62%) and antibody positivity (43.87% vs. 19.06%; both *p* < 0.001). The detection rates of nodules and cysts increased significantly with age, while antibody positivity decreased with advancing age.

**Conclusion:**

Thyroid abnormalities are highly prevalent in this health check-up population. Routine ultrasonography combined with a TSH-first screening strategy may facilitate early detection and risk stratification while balancing screening burden and cost-effectiveness. Particular attention should also be given to thyroid autoantibody screening, especially among young and middle-aged women.

## 1 Introduction

Thyroid diseases are among the most common endocrine disorders worldwide and can be broadly categorized into two types: structural abnormalities (such as thyroid nodules, cysts, and heterogeneous echotexture) and functional abnormalities (such as subclinical hypothyroidism, overt hypothyroidism, subclinical hyperthyroidism, and overt hyperthyroidism) (1–3). These disorders not only affect metabolism, cardiovascular and neurocognitive functions but also exert significant impacts on patients’ quality of life and public health burden (1, 4–7). Therefore, elucidating their epidemiological characteristics holds great clinical and public health significance (8).

Among structural thyroid abnormalities, thyroid nodules are the most extensively studied condition. Multiple studies have shown that the prevalence of thyroid nodules in the general population ranges from 20 to 76% (9–11). In a large-scale cross-sectional study conducted in Northeast China, the overall prevalence of thyroid nodules was 55.2% (9). At the global level, the incidence of thyroid diseases has shown an upward trend over the past two decades (12). In addition to nodules, thyroid cysts, heterogeneous echotexture, and parathyroid abnormalities are also common and clinically relevant structural disorders (3, 13).

Thyroid functional abnormalities are equally prevalent. These include both clinical and subclinical states, which exert profound systemic effects. Subclinical hypothyroidism (SCH) is the most common form of functional abnormality, with a prevalence ranging from 6.48 to 13.7% in the general population (14–16). According to large-scale cross-sectional data from China, the prevalence of SCH in adults is 13.93%, of which mild SCH (TSH 4.2–9.9 mIU/L) accounts for 12.18% and severe SCH (TSH ≥ 10 mIU/L) for 0.75% (17). The prevalence of overt hypothyroidism is approximately 1.02%, and thyroid autoantibodies (TPOAb and TGAb) are closely associated with thyroid dysfunction (17, 18). Regarding hyperthyroidism, the prevalence rates of overt hyperthyroidism (OH), Graves’ disease (GD), severe subclinical hyperthyroidism, and mild subclinical hyperthyroidism in mainland China are 0.78, 0.53, 0.22, and 0.22%, respectively (19). Evidence-based studies have shown that individuals with positive thyroid antibodies, particularly those with mildly elevated TSH, have a significantly increased risk of developing overt hypothyroidism in the future. Therefore, antibody testing plays an important role in risk stratification and follow-up decision-making in both clinical and population-based screening settings (20).

Although numerous studies have reported the epidemiological characteristics of thyroid nodules and thyroid functional abnormalities, most have been short-term and cross-sectional in nature, with limited evidence describing temporal trends across consecutive years. Moreover, many investigations have focused primarily on thyroid nodules and the three thyroid function parameters, with few providing a comprehensive assessment of structural thyroid abnormalities. In addition, the rising trend of autoimmune thyroid diseases in large populations has drawn increasing attention to thyroid antibody profiles (21). In recent years, thyroid function testing has been included as a basic component of adult health examinations in China, yet the justification for such extensive screening warrants further exploration. Furthermore, Dalian is a city with unique iodine nutrition profiles. Its natural environment—including soil and water—is deficient in iodine, yet it boasts abundant seafood resources with high dietary iodine content. Therefore, its iodine intake levels and thyroid disease prevalence have consistently attracted significant attention. However, studies based on large-sample data in Dalian remain limited, restricting the development and optimization of screening and follow-up strategies. Therefore, understanding the specific patterns of structural and functional thyroid abnormalities in Dalian is crucial for formulating targeted public health strategies and optimizing clinical management. Based on nearly 100,000 health check-up cases over the past 5 years, this study aims to fill the gap in regional epidemiological data, characterize the epidemiological patterns of thyroid abnormalities in Dalian, and provide a foundation for future studies evaluating targeted screening and prevention strategies.

## 2 Materials and methods

### 2.1 Study population

This study was designed as a retrospective cross-sectional study. A total of 103,640 adults who underwent thyroid ultrasonography at the Health Management Center of the First Affiliated Hospital of Dalian Medical University between January 2020 and December 2024 were initially enrolled (Figure 1). After excluding 3,661 individuals with a history of thyroid surgery, congenital thyroid absence, incomplete data, or repeated examinations, 99,979 participants were included in the thyroid ultrasonography analysis. Among those who underwent both thyroid ultrasonography and thyroid function testing, participants with incomplete thyroid function data were excluded, resulting in 17,768 subjects included in the analysis of thyroid function abnormalities. Of these, 8,278 individuals additionally underwent thyroid antibody testing. The study was approved by the Ethics Committee of the First Affiliated Hospital of Dalian Medical University (Approval No.: PJ-KS-KY-2025-977), which waived the requirement for informed consent.

**Figure 1:**
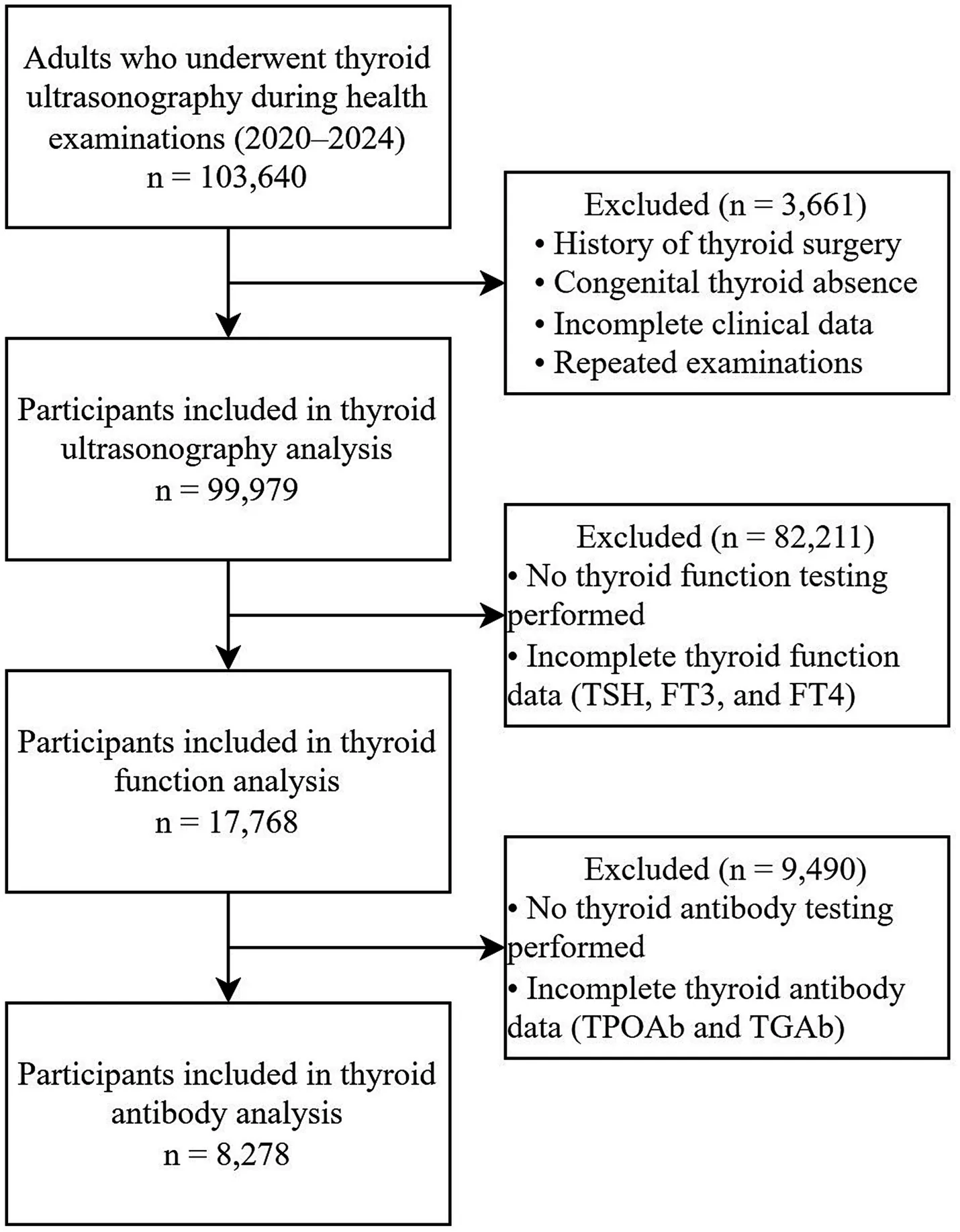
Flowchart of participant selection. Adults who underwent thyroid ultrasonography during health examinations at the Health Management Center of the First Affiliated Hospital of Dalian Medical University (2020–2024) were screened sequentially for inclusion in the thyroid ultrasonography, thyroid function, and thyroid antibody analyses. Participants were excluded at each stage for prior thyroid surgery, congenital thyroid absence, incomplete or repeated data, or missing thyroid function/antibody testing, as detailed in the figure.

### 2.2 Data collection and definition

Thyroid ultrasonography: Color Doppler ultrasound diagnostic system APLIO 400 TUS-A400 was used, with a high-frequency linear array probe operating at 10 MHz. Bilateral scanning of the thyroid lobes was performed. The examinee was placed in a supine position with the head tilted backward to fully expose the anterior neck region. The screening included assessment of thyroid nodule size, echogenicity, presence of calcification, margin clarity, vascular characteristics, and structural abnormalities such as suspected parathyroid lesions. The definitions were as follows:

Thyroid nodule: hypoechoic, isoechoic, hyperechoic, and mixed echogenicity.

Thyroid cyst: a cystic anechoic lesion within the thyroid gland.

Heterogeneous echotexture: non-uniform echogenicity within the thyroid parenchyma.

Suspected parathyroid abnormality: a hypoechoic mass located posterior to the dorsal side, upper, or lower pole of the thyroid gland.

Thyroid function testing: On the day of the health examination, 5 mL of fasting venous blood was collected. After coagulation, the sample was centrifuged at 3,000 r/min for 15 min. The serum was then used to measure TSH, FT3, and FT4 levels using the Mindray CL-6000i fully automated chemiluminescence immunoassay analyzer, and A-TG and A-TPO were measured using the Roche E411 fully automated chemiluminescence immunoassay analyzer. The reference ranges for thyroid function and antibody indicators were as follows:

Thyroid-stimulating hormone (TSH): 0.35–5.1 μIU/mL;

Free triiodothyronine (FT3): 2.76–6.45 pmol/L;

Free thyroxine (FT4): 11.2–23.81 pmol/L;

Anti-thyroglobulin antibody (A-TG): 0–115 IU/mL;

Anti-thyroid peroxidase antibody (A-TPO): 0–34 IU/mL.

### 2.3 Statistical analysis

All statistical analyses were performed using R software (version 4.2.2). The significance level was set at α = 0.05, and a two-sided *p* < 0.05 was considered statistically significant. For continuous variables, normality was assessed using histograms and the Shapiro–Wilk test. Normally distributed variables were expressed as mean ± standard deviation (Mean ± SD), and comparisons between groups were performed using the t-test or analysis of variance (ANOVA). Non-normally distributed variables were expressed as median (interquartile range, IQR) or, where appropriate, presented with mean, SD, median, IQR, minimum, and maximum values for comprehensive description. Group comparisons for non-normally distributed data were performed using the Wilcoxon rank-sum test (Mann–Whitney *U* test) or Kruskal–Wallis test. Categorical variables were expressed as frequencies (percentages), and comparisons between groups were performed using Fisher’s exact test when the expected frequency was less than 5; otherwise, Pearson’s chi-squared test was used. Prevalence estimates were reported with corresponding 95% confidence intervals (95% CIs), calculated using the Wilson score method.

## 3 Results

### 3.1 Study population

A total of 99,979 participants were included in the final analysis, all of whom completed thyroid ultrasonography. Among them, 17,768 participants underwent the three-item thyroid function test (TSH, FT3, and FT4), and 8,278 participants underwent the five-item thyroid function and antibody test (TSH, FT3, FT4, A-TG, and A-TPO). As shown in Table 1, the distribution of thyroid ultrasonography, thyroid function tests, and thyroid antibody testing in gender, age, and year was similar, but with a significant increase in thyroid function testing cases in 2024. Detailed comparisons of thyroid ultrasound abnormalities and thyroid function and antibody abnormalities by sex, age, and year from 2020 to 2024, as well as thyroid hormone and antibody levels by sex and age, are shown in Figures 2–4.

**Table 1:** Baseline characteristics of the study population (*n*, %).

Characteristic	Thyroid ultrasonography	Thyroid function	Thyroid function and autoantibody
Gender			
Male	48,338(48.35)	7,683(43.24)	3,621(43.74)
Female	51,641(51.65)	10,085(56.76)	4,657(56.26)
Age			
<30	7,987(7.99)	1,447(8.14)	580(7.01)
30–39	27,631(27.64)	4,896(27.56)	2,152(26.00)
40–49	22,204(22.21)	5,011(28.20)	2,626(31.72)
50–59	20,949(20.95)	3,974(22.37)	1,803(21.78)
60–69	12,931(12.93)	1,725(9.71)	790(9.54)
>=70	8,277(8.28)	715(4.02)	327(3.95)
Year			
2020	16,797(16.80)	1,423(8.01)	1,113(13.45)
2021	20,468(20.47)	2,632(14.81)	1,930(23.31)
2022	20,074(20.08)	3,493(19.66)	1,829(22.09)
2023	22,091(22.10)	3,737(21.03)	1,466(17.71)
2024	20,549(20.55)	6,483(36.49)	1,940(23.44)
Total	99,979(100.00)	17,768(100.00)	8,278(100.00)

**Figure 2:**
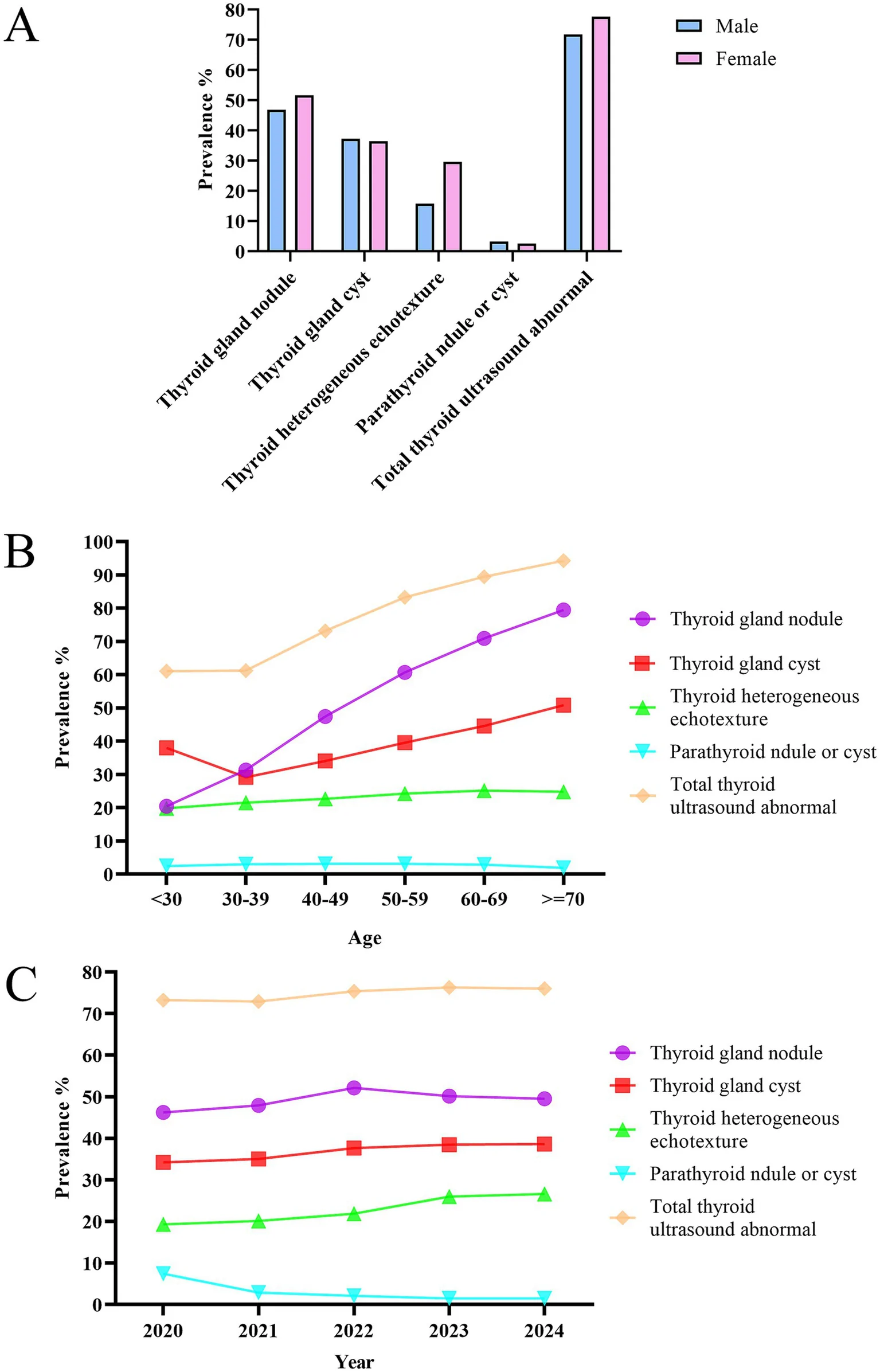
Prevalence of thyroid ultrasound abnormalities by sex, age, and year. **(A)** Comparison of thyroid ultrasound abnormalities between males and females. **(B)** Age-specific prevalence of thyroid gland nodule, thyroid gland cyst, thyroid heterogeneous echotexture, parathyroid nodule or cyst, and total thyroid ultrasound abnormalities. **(C)** Temporal trends in thyroid ultrasound abnormalities from 2020 to 2024.

**Figure 3:**
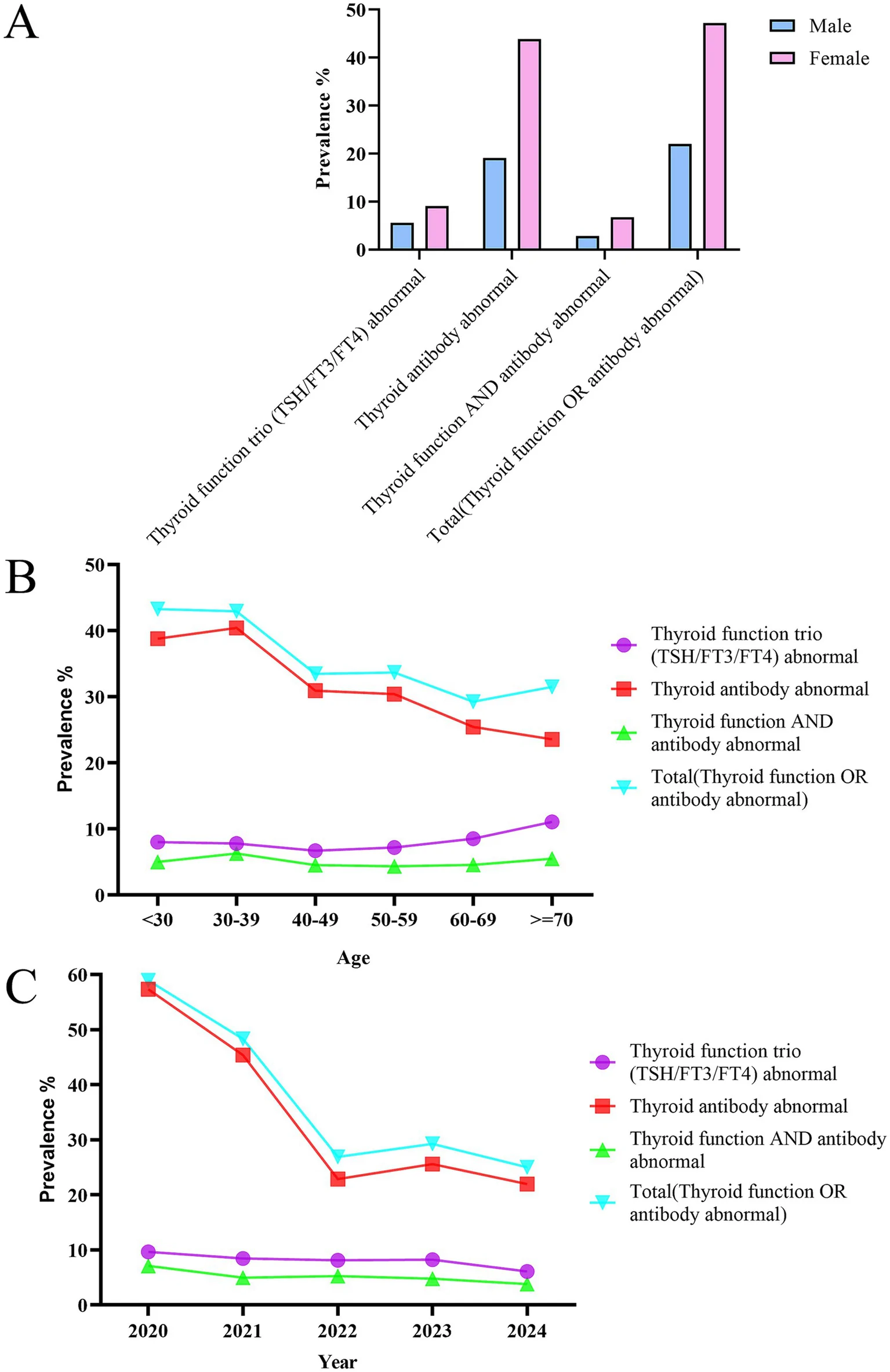
Prevalence of thyroid function and antibody abnormalities by sex, age, and year. **(A)** Comparison of thyroid function abnormalities (TSH/FT3/FT4), thyroid antibody abnormalities, combined thyroid function and antibody abnormalities, and total abnormalities between males and females. **(B)** Age-specific prevalence of thyroid function and antibody abnormalities. **(C)** Temporal trends in thyroid function and antibody abnormalities from 2020 to 2024.

**Figure 4:**
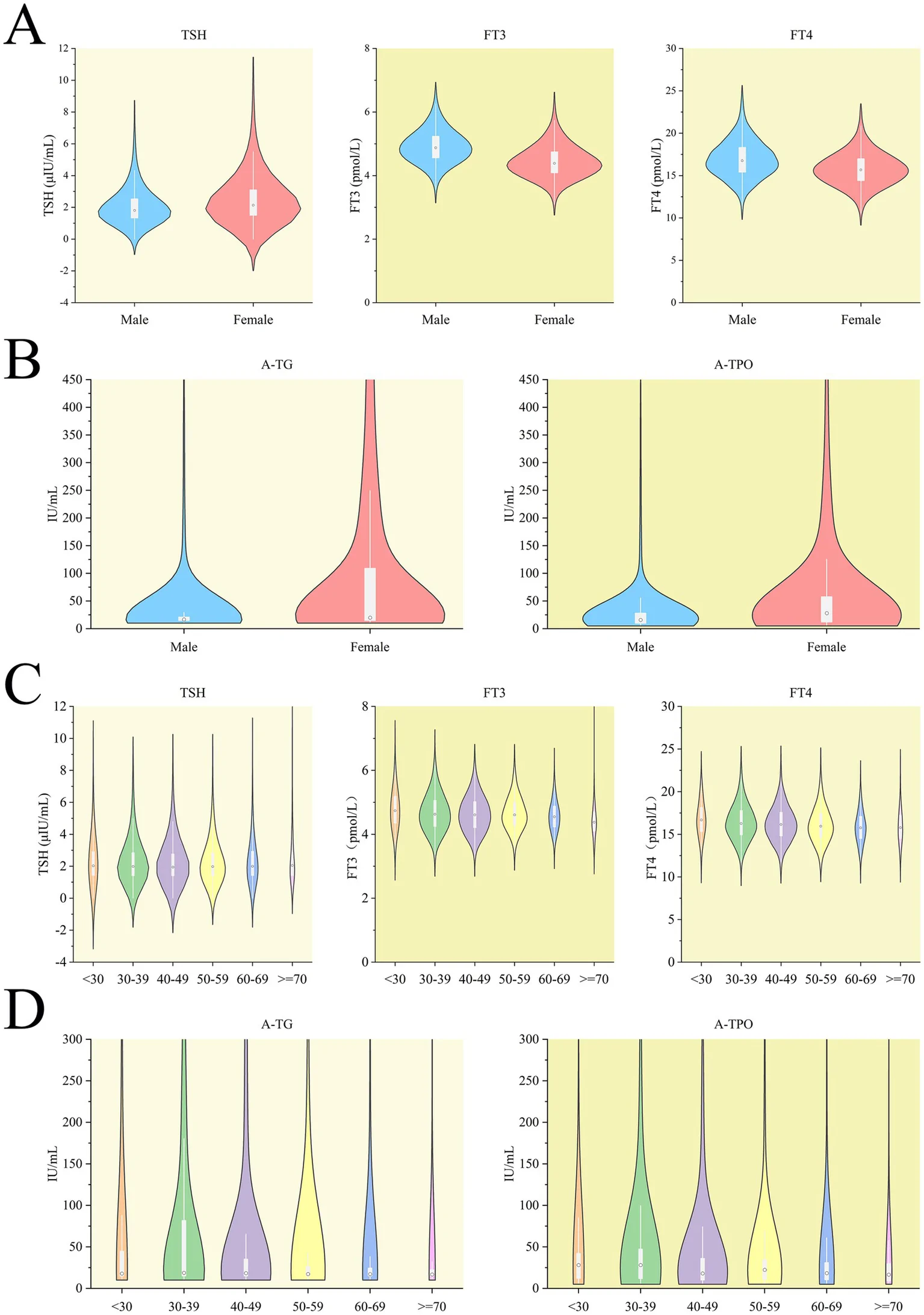
Distribution of thyroid hormone and antibody levels by sex and age. **(A)** Comparison of thyroid-stimulating hormone (TSH), free triiodothyronine (FT3), and free thyroxine (FT4) levels between females and males. **(B)** Comparison of thyroglobulin antibody (TGAb, A-TG) and thyroid peroxidase antibody (TPOAb, A-TPO) levels between females and males. **(C)** Age-specific distribution of TSH, FT3, and FT4 levels across different age groups. **(D)** Age-specific distribution of TGAb (A-TG) and TPOAb (A-TPO) levels across different age groups.

### 3.2 Thyroid ultrasonography findings

Among the 99,979 individuals who underwent thyroid ultrasonography, a total of 74,832 subjects were found to have thyroid abnormalities, accounting for 74.85% (95% CI: 74.58–75.12%). Among all types of abnormalities, thyroid nodules were the most common (49.29, 95% CI: 48.98–49.60%), followed by thyroid cysts (36.91, 95% CI: 36.61–37.21%) and heterogeneous echotexture (22.96, 95% CI: 22.70–23.22%), whereas the detection rate of parathyroid nodules or cysts was relatively low (2.91, 95% CI: 2.81–3.01%). The detection rate of most types of thyroid abnormalities was higher in females than in males, with the most significant difference observed in heterogeneous echotexture (females 29.61% vs. males 15.85%, *p* < 0.001). The overall detection rate of abnormalities was 77.72% in females and 71.78% in males. The detection rates of thyroid nodules (females 51.60% vs. males 46.82%), thyroid cysts (females 36.53% vs. males 37.32%), and parathyroid nodules or cysts (females 2.63% vs. males 3.20%) were generally comparable between the two sexes.

Across different age groups, the detection rate of thyroid abnormalities increased with age, rising from 61.06% among individuals under 30 years to 94.25% among those aged 70 years and older. The detection rate of thyroid nodules increased from 20.46 to 79.45%, and that of thyroid cysts from 29.16% (30–39 years) to 50.82% (≥70 years), both showing a clear age-related upward trend. The detection rate of heterogeneous echotexture also showed a gradual increase with age (*p* < 0.001), though the overall rate remained approximately 20% across groups.

From a temporal perspective, between 2020 and 2024, the overall detection rate of thyroid abnormalities exhibited a slow upward trend, increasing from 73.25% in 2020 to 76.29% in 2023, with a slight decline to 76.00% in 2024. The detection rates of thyroid nodules, cysts, and heterogeneous echotexture all showed a gradual year-by-year increase, while parathyroid lesions demonstrated a marked downward trend (7.43% in 2020 to 1.50% in 2024, *p* < 0.001). Overall, thyroid nodules were the most common abnormality, and their detection rate showed statistically significant differences across gender, age, and year groups (all *p* < 0.001). Details are presented in Table 2.

**Table 2:** Detection of thyroid ultrasound abnormalities across different genders, age groups and years (*n*, %).

Characteristic	Thyroid gland nodule	Thyroid gland cyst	Thyroid heterogeneous echotexture	Parathyroid nodule or cyst	Total thyroid ultrasound abnormality
Gender					
Male	22,630 (46.82)	18,039 (37.32)	7,663 (15.85)	1,547 (3.20)	34,695 (71.78)
Female	26,648 (51.60)	18,865 (36.53)	15,292 (29.61)	1,360 (2.63)	40,137 (77.72)
Statistics	228.83	6.65	2,672.23	28.41	469.09
*p*-value	<0.001	0.01	<0.001	<0.001	<0.001
Age					
<30	1,634 (20.46)	3,036 (38.01)	1,583 (19.82)	198 (2.48)	4,877 (61.06)
30–39	8,659 (31.34)	8,057 (29.16)	5,949 (21.53)	822 (2.97)	16,913 (61.21)
40–49	10,528 (47.41)	7,554 (34.02)	5,039 (22.69)	696 (3.13)	16,237 (73.13)
50–59	12,712 (60.68)	8,289 (39.57)	5,076 (24.23)	658 (3.14)	17,439 (83.25)
60–69	9,169 (70.91)	5,762 (44.56)	3,256 (25.18)	374 (2.89)	11,565 (89.44)
>=70	6,576 (79.45)	4,206 (50.82)	2,052 (24.79)	159 (1.92)	7,801 (94.25)
Statistics	12,767.17	1,872.30	148.18	42.28	7,472.37
*p*-value	<0.001	<0.001	<0.001	<0.001	<0.001
Year					
2020	7,761 (46.20)	5,747 (34.21)	3,240 (19.29)	1,248 (7.43)	12,304 (73.25)
2021	9,808 (47.92)	7,173 (35.04)	4,116 (20.11)	592 (2.89)	14,926 (72.92)
2022	10,460 (52.11)	7,555 (37.64)	4,393 (21.88)	426 (2.12)	15,131 (75.38)
2023	11,078 (50.15)	8,498 (38.47)	5,738 (25.97)	332 (1.50)	16,854 (76.29)
2024	10,171 (49.50)	7,931 (38.60)	5,468 (26.61)	309 (1.50)	15,617 (76.00)
Statistics	149.95	135.63	503.34	1,558.58	104.96
*p*-value	<0.001	<0.001	<0.001	<0.001	<0.001
Total	49,278 (49.29)	36,904 (36.91)	22,955 (22.96)	2,907 (2.91)	74,832 (74.85)

### 3.3 Thyroid function and antibody findings

Among the 17,768 participants who underwent thyroid function testing and the 8,278 participants who underwent thyroid antibody testing, the overall detection rate of abnormalities in the three thyroid function parameters (TSH/FT3/FT4) was 7.57% (95% CI: 7.19–7.97%). The detection rate of thyroid antibody abnormalities (TPOAb and TGAb) was 33.02% (95% CI: 32.01–34.04%), and the proportion of individuals with both thyroid function and antibody abnormalities was 5.01% (95% CI: 4.56–5.50%). The overall prevalence of any abnormality (function or antibody) was 36.18% (95% CI: 35.15–37.22%). Among the functional abnormalities, the detection rates for overt hyperthyroidism, subclinical hyperthyroidism, overt hypothyroidism, and subclinical hypothyroidism were 0.61% (*N* = 108), 0.79% (*N* = 140), 0.42% (*N* = 75), and 4.69% (*N* = 834), respectively. Abnormalities involving TSH were predominant: the overall rate of TSH abnormalities accounted for 86.32% (1,161/1,345) of all functional abnormalities, of which isolated TSH abnormalities accounted for 72.42% (974/1,345).

Females showed significantly higher detection rates of thyroid function and antibody abnormalities than males, with the most pronounced difference observed in thyroid antibody positivity, which was more than twice that of males (43.87% vs. 19.06%, *p* < 0.001). The rates of abnormalities in thyroid function and combined function-antibody abnormalities were also significantly higher in females than in males (9.05% vs. 5.62, and 6.72% vs. 2.82%, respectively; both *p* < 0.001).

Regarding age, the detection rate of antibody abnormalities was higher among participants under 30 years (38.79%) and those aged 30–39 years (40.43%), and showed a decreasing trend with increasing age, reaching the lowest rate of 23.55% in individuals aged 70 years and older, with statistically significant differences across age groups (*p* < 0.001). However, the prevalence of abnormalities in the three thyroid function parameters differed significantly among age groups (*p* < 0.001), while combined function-antibody abnormalities showed no significant differences, ranging from 6.71–11.05% and 4.33–6.27%, respectively.

From an annual perspective, thyroid antibody abnormalities exhibited remarkably high detection rates in 2020 and 2021, followed by a significant year-by-year decline, dropping from 57.32% in 2020 to 21.96% in 2024 (*p* < 0.001). The annual variation in thyroid function abnormalities was relatively small, showing a slight downward trend overall (9.63% in 2020 to 6.08% in 2024). The proportion of individuals with both function and antibody abnormalities also showed a gradual decline during the same period. Details are shown in Tables 3, 4.

**Table 3:** Detection of abnormal thyroid function and antibodies across different genders, age groups, and years (*n*, %).

Characteristic	Thyroid function trio (TSH/FT3/FT4) abnormality	Thyroid antibody abnormality	Thyroid function AND antibody abnormality	Total (thyroid function OR antibody abnormality)
Gender				
Male	432 (5.62)	690 (19.06)	102 (2.82)	797 (22.01)
Female	913 (9.05)	2,043 (43.87)	313 (6.72)	2,198 (47.20)
Statistics	73.34	567.17	65.20	559.69
*p*-value	<0.001	<0.001	<0.001	<0.001
Age				
<30	116 (8.02)	225 (38.79)	29 (5.00)	251 (43.28)
30–39	381 (7.78)	870 (40.43)	135 (6.27)	924 (42.94)
40–49	336 (6.71)	812 (30.92)	119 (4.53)	879 (33.47)
50–59	286 (7.20)	548 (30.39)	78 (4.33)	607 (33.67)
60–69	147 (8.52)	201 (25.44)	36 (4.56)	231 (29.24)
>=70	79 (11.05)	77 (23.55)	18 (5.50)	103 (31.50)
Statistics	21.47	106.76	10.75	88.05
*p*-value	<0.001	<0.001	0.057	<0.001
Year				
2020	137 (9.63)	638 (57.32)	79 (7.10)	656 (58.94)
2021	222 (8.43)	876 (45.39)	96 (4.97)	933 (48.34)
2022	284 (8.13)	418 (22.85)	96 (5.25)	492 (26.90)
2023	308 (8.24)	375 (25.58)	70 (4.77)	429 (29.26)
2024	394 (6.08)	426 (21.96)	74 (3.81)	485 (25.00)
Statistics	38.04	660.25	16.41	576.93
*p*-value	<0.001	<0.001	0.003	<0.001
Total	1,345 (7.57)	2,733 (33.02)	415 (5.01)	2,995 (36.18)

**Table 4:** Specific detection of thyroid function abnormalities (*n*, %).

Characteristic	Total	Gender	
		Male	Female
Abnormal TSH combined with abnormal FT3/FT4	187(1.05)	66(0.37)	121(0.68)
TSH↑ with FT3/FT4↓ (Hypothyroidism)	75(0.42)	22(0.12)	53(0.30)
TSH↓ with FT3/FT4↑ (Hyperthyroidism)	108(0.61)	41(0.23)	67(0.38)
TSH↑ with FT3/FT4↑	2(0.01)	2(0.01)	0(0.00)
TSH↓ with FT3/FT4↓	2(0.01)	1(0.00)	1(0.01)
Isolated Abnormal TSH	974(5.48)	263(1.48)	711(4.00)
TSH↑ (Subclinical Hypothyroidism)	834(4.69)	228(1.28)	606(3.41)
TSH↓ (Subclinical Hyperthyroidism)	140(0.79)	35(0.20)	105(0.59)
Isolated Abnormal FT3 or FT4	179(1.01)	100(0.56)	79(0.44)
FT3	67(0.38)	48(0.27)	19(0.11)
FT4	112(0.63)	52(0.29)	60(0.34)
Isolated Abnormal FT3 and FT4	5(0.03)	3(0.02)	2(0.01)

A detailed analysis of hormonal and antibody levels revealed that the mean FT3 and FT4 levels decreased progressively with age, and that female FT3 and FT4 levels were consistently lower than those of males across all age groups. The mean TSH level remained relatively stable, though slightly higher in females than in males. Regarding antibodies, mean TPOAb and TGAb levels were generally higher in females than in males, with the most pronounced differences observed in younger populations (e.g., TPOAb: 141 ± 331 in females <30 years vs. 36 ± 116 in males). Details are presented in Table 5.

**Table 5:** Results of the three thyroid function parameters and thyroid antibody levels by gender and age group.

Age	TSH		FT3		FT4		TPOAb		TGAb	
	Male	Female	Male	Female	Male	Female	Male	Female	Male	Female
<30	2.17 ± 1.36	2.90 ± 7.86	5.23 ± 1.54	4.70 ± 1.67	18.03 ± 3.50	16.40 ± 3.26	36 ± 116	141 ± 331	30 ± 93	127 ± 364
30–39	2.07 ± 1.42	2.74 ± 6.09	5.13 ± 1.18	4.53 ± 1.11	17.54 ± 2.50	16.04 ± 3.18	60 ± 180	167 ± 350	52 ± 159	124 ± 318
40–49	2.36 ± 5.10	2.75 ± 6.62	5.03 ± 1.20	4.38 ± 0.81	17.18 ± 3.13	15.62 ± 2.51	53 ± 175	136 ± 314	60 ± 245	122 ± 312
50–59	2.22 ± 2.85	2.87 ± 5.95	4.89 ± 0.76	4.49 ± 0.94	16.74 ± 2.99	15.67 ± 2.70	46 ± 157	144 ± 320	42 ± 207	135 ± 441
60–69	2.47 ± 5.26	2.76 ± 3.35	4.74 ± 0.68	4.47 ± 0.69	16.11 ± 2.43	15.71 ± 2.32	41 ± 137	143 ± 328	60 ± 307	104 ± 228
> = 70	2.60 ± 1.93	2.87 ± 2.89	4.47 ± 0.53	4.31 ± 0.56	16.23 ± 3.29	15.78 ± 2.46	40 ± 117	91 ± 253	41 ± 154	154 ± 356
Statistics	32.42	14.08	702.36	200.93	437.86	132.47	50.74	10.96	14.5	6.98
*p*-value	<0.001	0.015	<0.001	<0.001	<0.001	<0.001	<0.001	0.052	0.013	0.222

### 3.4 Analysis of abnormal thyroid function combined with abnormal thyroid antibody conditions

Among the 8,278 participants who completed both thyroid function and antibody testing, the overall positivity rate of TPOAb was 28.09%, which was substantially higher than that of TGAb (16.32%). This demonstrates that TPOAb is the dominant autoantibody in this population. In addition, 11.39% were positive for both TPOAb and TGAb, 16.69% were positive for TPOAb only, 4.93% were positive for TGAb only, and 66.98% were negative for both antibodies.

Among all antibody-positive individuals, those with TPOAb(+)/TGAb(+) had the highest rate of thyroid function abnormalities (24.60%), followed by the TPOAb(+)/TGAb(−) group (10.93%) and the TPOAb(−)/TGAb(+) group (7.84%), while the double antibody-negative group had the lowest rate of thyroid function abnormalities (4.72%). Details are presented in Table 6.

**Table 6:** Analysis of thyroid function abnormalities combined with thyroid antibody abnormalities (*n*, %).

Characteristic	Total number of cases	Thyroid function trio (TSH/FT3/FT4) status	
		Normal	Abnormal
TPOAb(+)TGAb(+)	943 (11.39)	711 (75.40)	232 (24.60)
TPOAb(+)TGAb(−)	1,382 (16.69)	1,231 (89.07)	151 (10.93)
TPOAb(−)TGAb(+)	408 (4.93)	376 (92.16)	32 (7.84)
TPOAb(−)TGAb(−)	5,545 (66.98)	5,283 (95.28)	262 (4.72)

## 4 Discussion

A comprehensive retrospective analysis was conducted on thyroid ultrasonographic characteristics and thyroid function among individuals undergoing health examinations between 2020 and 2024. Overall, thyroid structural abnormalities were highly prevalent, particularly thyroid nodules. Thyroid function abnormalities were predominantly characterized by hypothyroidism, whereas thyroid autoantibody positivity was common and showed a close association with thyroid dysfunction. Distinct sex-, age-, and time-related patterns were observed, providing epidemiological evidence that may inform future evaluation of risk-stratified thyroid screening strategies. In addition, the transient increase in antibody positivity during 2020–2021 generated the hypothesis that pandemic-related immune stimulation may have contributed to this observation. However, this remains a hypothesis and lacks relationship analysis.

### 4.1 Thyroid ultrasonography

Our study revealed that the prevalence of thyroid ultrasonographic abnormalities among the health examination population in Dalian was as high as 74.85%, predominantly due to thyroid nodules (49.29%). This prevalence is consistent with recent trends reported in large-scale screening studies utilizing high-frequency ultrasonography. For example, the detection rate of thyroid nodules among adults in Zhejiang Province was 50.98% (22). However, it was higher than that reported in several other regions of China, such as Hangzhou, where the prevalence among males and females was 24.1 and 34.7%, respectively (23). In Chongqing, the prevalence of thyroid nodules detected during health examinations was 34.1% (24), while in Tianjin, the prevalence among adults was 40.00% (25). The higher detection rate of thyroid nodules in Dalian may be attributed to several factors. Dalian is a city with unique iodine nutrition profiles. Although it is a coastal city, its natural environment—including soil and water—is deficient in iodine, yet it boasts abundant seafood resources with high dietary iodine content. As a coastal city, iodine intake in Dalian may fluctuate with iodized-salt use and seafood consumption. Chronic iodine excess can induce thyroid cell proliferation, increase thyroid-stimulating hormone (TSH) sensitivity, and subsequently promote nodule formation. This finding is consistent with the established “U-shaped” relationship between iodine intake and thyroid diseases. That is, both iodine deficiency and iodine excess can lead to thyroid dysfunction and autoimmune thyroid diseases. Iodine deficiency can result in goiter and hypothyroidism, while iodine excess may cause autoimmune thyroid dysfunction (26–29). However, as individual iodine status was not directly measured in this study, iodine excess remains only one of several plausible explanations, and other unmeasured factors such as BMI, smoking, medication use, family history, metabolic status, and socioeconomic conditions may also contribute and cannot be excluded. Meanwhile, the widespread application of high-resolution ultrasonography has markedly improved the detection of subclinical nodules, partially explaining the upward trend in thyroid nodule prevalence observed in recent studies (30–32).

Our study found that the detection rate of thyroid cysts among the health check-up population in Dalian was relatively high (36.91%), with 18,039 males (37.32%) and 18,865 females (36.53%) affected, closely resembling findings from Japan. A Japanese study involving 1,432 participants reported an overall prevalence of thyroid cysts of 32.8%, with 27.4% in males and 35.9% in females (33). This may collectively reflect a regional characteristic of a high prevalence of thyroid cystic lesions in East Asian coastal populations. This relatively high detection rate may be associated with multiple contributing factors, including natural degenerative changes in thyroid follicular structures with aging, iodine intake in coastal regions (which may fluctuate with iodized-salt use and seafood consumption), and other possible factors such as BMI and metabolic status (8, 34–37).

Heterogeneous echotexture of the thyroid parenchyma is a typical ultrasonographic feature of autoimmune thyroiditis, reflecting the underlying pathology of Hashimoto’s thyroiditis, including lymphocytic infiltration, follicular cell destruction, and fibrosis, and is more common in females (38, 39). Our study found a relatively high detection rate of heterogeneous echotexture in Dalian (22.96%), strongly suggesting a substantial burden of autoimmune thyroid disease in this population.

In sharp contrast to the high detection rates of nodules, cysts, and heterogeneous echotexture, the detection rate of suspected parathyroid abnormalities declined markedly from 7.43% in 2020 to 1.50% in 2024. The reason for this decline remains unclear and may be related to changes in ultrasound techniques or diagnostic proficiency, warranting further investigation in future studies.

### 4.2 Thyroid function and antibodies

This study systematically analyzed thyroid function among 17,768 individuals who underwent health examinations and found an overall abnormal rate of 7.57%. Among these, hypothyroidism (including subclinical and overt types) was the most prevalent abnormality, with a total prevalence of 5.11% (subclinical hypothyroidism 4.69%, overt hypothyroidism 0.42%); hyperthyroidism (including subclinical and overt types) had a total prevalence of 1.40% (subclinical hyperthyroidism 0.79%, overt hyperthyroidism 0.61%); and other abnormalities accounted for 188 cases (1.06%). From an epidemiological perspective, functional abnormalities were significantly more common in females (9.05%) than in males (5.62%) (*p* < 0.001), and the prevalence of thyroid dysfunction increased non-linearly with age, rising from 8.02% in individuals younger than 30 years to 11.05% in those aged 70 years or older.

Compared with other large-scale epidemiological surveys in China, the overall prevalence of hypothyroidism (5.11%) observed in this study was lower. A large national survey covering 31 provinces in China reported that between 2015 and 2017, the overall prevalence of hypothyroidism (including subclinical) among Chinese adults was approximately 13.95%, of which overt hypothyroidism accounted for 1.02%, while subclinical hypothyroidism (mainly mild forms) constituted the majority (~12.18%) (17). This discrepancy may result from differences in population representativeness and demographic structure: First, this study was based on hospital-based health examination participants, who may exhibit selection bias (e.g., greater health awareness) compared with the general community population. Second, differences in age distribution likely played a role; the average age of the population in the referenced national survey was slightly higher than that of our study population. Given that the prevalence of hypothyroidism is positively correlated with age, this younger demographic profile may contribute to the lower prevalence observed in our study. One study reported that in mainland China, the prevalence of overt hyperthyroidism (OH), Graves’ disease (GD), severe subclinical hyperthyroidism (severe SCH), and mild subclinical hyperthyroidism (mild SCH) was 0.78, 0.53, 0.22, and 0.22%, respectively (19). The increasing prevalence of thyroid dysfunction with age—particularly the rise in subclinical hypothyroidism—reflects the cumulative effect of physiological decline in thyroid function associated with aging.

One of the most striking findings of this study was the high prevalence (33.02%) of thyroid autoantibody positivity (TPOAb or TGAb) among the health examination population in Dalian. This rate was particularly elevated in females (43.87%), more than double that in males (19.06%) (*p* < 0.001). In contrast to the age-related increase observed in thyroid functional abnormalities, antibody positivity exhibited a declining trend with age, peaking in the 30–39-year age group (40.43%) and reaching the lowest level (23.55%) in individuals aged ≥70 years. The antibody positivity rate observed in this study (33.02%) was significantly higher than that reported in several large-scale national studies. For instance, a study involving 78,470 participants aged ≥18 years from 31 provinces across mainland China reported positivity rates of 14.19% for any thyroid antibody, 10.19% for thyroid peroxidase antibody (TPOAb), and 9.70% for thyroglobulin antibody (TGAb) (40). Another study conducted in Qinghai Province between September 2015 and March 2016 involving 2,628 healthy urban residents reported TPOAb and TGAb positivity rates of 9.80 and 9.20%, respectively (41). This pronounced regional discrepancy may be attributed to gene-environment interactions. Certain genetic polymorphisms may enhance the immunogenicity of thyroglobulin molecules via oxidative modification. Iodine intake in coastal regions, which may fluctuate with iodized-salt use and seafood consumption, represents one possible environmental factor, as excessive iodine can thereby disrupt immune tolerance in genetically susceptible individuals (42). As individual iodine status was not measured in this study, this is just a speculative explanation. Moreover, compared with previous population-based cross-sectional surveys, our retrospective design and sample selection might also have contributed to the higher observed prevalence.

Another key finding of this study was the clear association between antibody status and the risk of thyroid dysfunction. Compared with individuals negative for both antibodies (thyroid dysfunction rate 4.72%), those positive only for TGAb [TPOAb(−)TGAb(+)] showed a slightly higher dysfunction rate of 7.84%, while those positive only for TPOAb [TPOAb(+)TGAb(−)] exhibited a markedly higher risk (10.93%). This risk peaked among individuals positive for both antibodies [TPOAb(+)TGAb(+)], whose thyroid dysfunction rate reached 24.60%, more than five times that of the double-negative group.

Furthermore, our findings indicate that abnormalities involving TSH accounted for the majority of thyroid function abnormalities in this health check-up population. The overall functional abnormality rate was a relatively moderate 7.57% (*N* = 1,345) and was overwhelmingly dominated by TSH-related issues. Abnormalities involving TSH accounted for 86.32% (1,161/1,345) of all functional abnormalities, with isolated TSH abnormalities alone accounting for 72.42% (974/1,345). The current common practice in China’s health check-up system often involves universal screening with the full three-item panel (TSH, FT3, and FT4). However, given that most thyroid function abnormalities involved TSH, our findings raise the hypothesis that a TSH-first testing approach may be more efficient, which is consistent with several other clinical settings (43, 44).

### 4.3 Correlation between gender and thyroid diseases

The correlation between gender and thyroid diseases shows that women have a heightened predisposition to autoimmune thyroid diseases and thyroid nodules (45, 46). Women not only show a greater prevalence of thyroid nodules than men (9, 10), but also a higher incidence of autoimmune thyroid diseases (17). Research uniformly indicates that females are disproportionately burdened by thyroid disorders. This study of a health check-up population in Dalian found that the prevalences of both ultrasound abnormalities (77.72%) and nodules (51.60%) were slightly higher in females compared to males (71.78 and 46.82%, respectively). Additionally, the detection rate for heterogeneous thyroid parenchyma was significantly higher in women (29.61% vs. 15.85%). Females also exhibited higher rates of thyroid function abnormalities (9.05% vs. 5.62%) and antibody positivity (43.87% vs. 19.06%). The reasons for this female susceptibility to thyroid nodules and autoimmune thyroid diseases are multifaceted. Studies indicate that estrogen exerts a proliferative effect on thyroid cells. Estrogen stimulates thyroid follicular growth and vascular distribution, thereby increasing the likelihood of nodular changes in women. It also enhances the production of autoantibodies and increases thyroid sensitivity. Concurrently, the complexity of X chromosome inactivation and the regulatory effects of sex hormones on T-cell and B-cell function collectively create a pro-inflammatory environment, reducing the onset threshold for autoimmune diseases. Additionally, estrogen, estrogen receptors, and estrogen receptor-mediated signaling pathways may specifically contribute to the gender disparity in autoimmune thyroid disease (AITD) by influencing the number and function of immune cells as well as thyroid homeostasis (47).

Notably, this study found that the thyroid function abnormality rate (5.62%) and the antibody abnormality rate (19.06%) in males are also relatively high, with the antibody abnormality rate exceeding the national average level of 14.1% (48). This suggests that the prevalence of elevated thyroid antibody abnormalities in the overall Dalian population warrants further attention. This high prevalence trend observed in both women and the general male population suggests that besides the influence of sex hormones, there are likely more widespread shared risk factors, such as regional iodine nutrition status (26–28), genetic susceptibility (49), and the rising prevalence of obesity and metabolic syndrome (50).

### 4.4 Correlation between age and thyroid diseases

Age is another key factor influencing thyroid disorders; however, its effects on structural and functional abnormalities of the thyroid differ markedly (51). In this study, the detection rate of thyroid ultrasound abnormalities increased significantly with age (total abnormality rate rising from 61.06 to 94.25%). The prevalence of thyroid nodules and cysts showed an almost linear upward trend with increasing age — the nodule detection rate increased from 20.46% in individuals under 30 years old to 79.45% in those aged 70 years or older, while the cyst detection rate rose from 29.16% (30–39 years) to 50.82% (≥70 years). The detection rate of heterogeneous thyroid echotexture. The detection rate of heterogeneous thyroid echotexture increased slightly from 19.82 to 24.79%. These findings are consistent with previous studies, which have demonstrated that the prevalence of thyroid nodular disease rises with age. One study reported that between 1995 and 2011, the number of nodules detected at first diagnosis increased from an average of 1.5 in the youngest group (aged 20–30 years) to 2.2 in the oldest group (≥70 years) (*p* < 0.001), with a 1.6% annual increase in multinodularity (odds ratio, 1.02; *p* < 0.001) (52).

This pattern can be attributed to the fact that once structural abnormalities such as nodules and cysts develop, they rarely regress. It also reflects the cumulative effects of lifelong somatic mutations (e.g., mutations in the TSH receptor gene), continuous proliferative stimuli, and cellular senescence (34). These factors impair the thyroid’s repair mechanisms and, with advancing age, lead to degenerative fibrosis and cyst formation. Accumulated somatic mutations and environmental exposures disrupt thyroid homeostasis, while immunosenescence weakens regulatory T-cell function, promoting nodule formation. In addition, chronic inflammation-induced progressive fibrosis and follicular atrophy contribute to degenerative cyst formation, leading to heterogeneous echogenicity. Age-related vascular changes and cumulative oxidative stress also contribute to this echogenic heterogeneity.

In contrast, autoimmune thyroid diseases (characterized by antibody positivity) exhibited a distinct age-related pattern: antibody positivity peaked in the 30–39-year age group and then gradually declined (from 38.79 to 23.55%). This trend aligns with the natural course of Hashimoto’s thyroiditis, which manifests as an active immune response in younger individuals that progressively induces thyroid follicular apoptosis. In older individuals, as thyroid tissue is destroyed, the autoimmune process may enter an “exhaustion phase,” during which decreased antigenic stimulation leads to lower antibody levels. However, because thyroid function is already severely impaired at this stage, the risk of clinical hypothyroidism continues to rise. Furthermore, a slight increase in functional abnormalities with age (from 8.02% in individuals under 30 to 11.05% in those aged ≥70 years) reflects non-immunological factors, such as age-related declines in hormone synthesis and vascular alterations. Reduced efficiency in thyroid hormone synthesis further exacerbates functional decline.

### 4.5 Prevalence trends of thyroid diseases from 2020 to 2024

From 2020 to 2024, the detection rates of thyroid structural abnormalities, including nodules (46.20 to 49.50%), cysts (34.21 to 38.60%), heterogeneous echotexture (19.29 to 26.61%), and overall abnormalities (73.25 to 76.00%), showed a gradual increase, while the detection rate of parathyroid lesions (7.43 to 1.50%) demonstrated a decline. The rising trend in thyroid structural abnormalities may be attributed to multiple factors, such as improvements in diagnostic methods, aging of the health examination population, and changes in environmental exposures, including pollution, which may exacerbate structural alterations over time. These findings also suggest that the clinical burden of thyroid diseases continues to increase.

In contrast, the decreasing detection of parathyroid lesions warrants cautious interpretation. Parathyroid cysts are rare, often nonfunctional, and frequently asymptomatic, making them difficult to distinguish from thyroid lesions in routine ultrasonography (53). Fewer than 300 cases had been reported worldwide by 2016, accounting for only 0.08–3.41% of all parathyroid lesions (54–56). Ultrasonography has been applied to detect parathyroid nodules in specific populations, such as patients with chronic kidney disease complicated by severe secondary hyperparathyroidism (57). Additionally, a longitudinal study in China reported a rising proportion of asymptomatic primary hyperparathyroidism between 2000 and 2010, largely driven by incidental findings on imaging (58). Therefore, temporal fluctuations in detection rates may partly reflect differences in diagnostic awareness, imaging practices, and case ascertainment rather than true epidemiological shifts.

From 2020 to 2024, the detection rates of thyroid function abnormalities (9.63 to 6.08%) and antibody positivity (57.32 to 21.96%) both showed a downward trend. Notably, the thyroid antibody positivity rate reached an exceptionally high level of 57.32% in 2020, then declined to a stable baseline of approximately 22% from 2022 onward. One possible hypothesis is that this transient increase may relate to SARS-CoV-2 infection and/or COVID-19 vaccination during the pandemic period. Recent studies on SARS-CoV-2 infection and COVID-19 vaccination suggest that infection or immune activation may cause short-term changes in thyroid function and antibody titers; however, most follow-up studies indicate that such abnormalities are generally reversible, and vaccination has not been associated with a significant outbreak of clinically apparent thyroid diseases (59). However, because individual-level information regarding SARS-CoV-2 infection and COVID-19 vaccination was unavailable, this interpretation should be regarded as a hypothesis generated from an ecological temporal association rather than evidence of a causal relationship. Therefore, when interpreting annual or short-term fluctuations in detection rates, potential influences from the pandemic/vaccination, changes in the composition of the screened population, and other unmeasured factors should also be considered (60).

### 4.6 Limitations

Several inherent limitations should be considered when interpreting the results of this study. First, this was a single-center retrospective study, and the study population consisted of individuals who voluntarily participated in health examinations. Compared with the general community population, these individuals may have higher health awareness and different socioeconomic backgrounds, resulting in potential selection bias. These attendees may also differ from the general population in healthcare access and health-seeking behavior, further limiting generalizability. Therefore, caution should be exercised when extrapolating the findings of this study to the general population of Dalian. Second, the retrospective nature of data collection limits our ability to infer causality. For example, we observed an abnormally increased thyroid antibody positivity rate during 2020–2021 and speculated that it might be related to the COVID-19 pandemic; however, this is a clinical speculation, lacking further investigation and analysis.

In addition, data completeness presents another challenge. Not all participants who underwent thyroid ultrasonography also completed thyroid function and antibody testing. This incomplete pairing may introduce bias when analyzing the prevalence of functional and antibody abnormalities. Furthermore, we were unable to obtain other important potential confounding factors, such as individual iodine nutritional status (e.g., urinary iodine levels), body mass index (BMI), smoking history, and detailed medication history, all of which may influence thyroid health.

Future studies should focus on conducting multicenter, prospective cohort investigations using standardized diagnostic and testing procedures, while collecting more comprehensive covariate data to validate the findings of this study.

## 5 Conclusion

This study, based on retrospective data from nearly 100,000 health examination participants over a five-year period in Dalian, China, systematically described the extremely high prevalence and complex epidemiological characteristics of thyroid abnormalities in this region. The study showed that structural thyroid abnormalities (predominantly thyroid nodules) and autoimmune thyroiditis (characterized by a high antibody positivity rate) represent the major thyroid health issues in Dalian. These abnormalities demonstrate significant heterogeneity in gender and age distribution, with females and older adults identified as the primary high-risk groups for thyroid diseases. Furthermore, participants with positive thyroid autoantibodies exhibited a significantly higher prevalence of thyroid dysfunction, suggesting that these autoantibodies might be a major contributing factor to the condition.

In summary, our findings suggest that the current “one-size-fits-all” approach to thyroid function screening may warrant further evaluation. We propose that future studies consider developing a risk prediction model for thyroid function and antibody abnormalities, with corresponding screening strategies—potentially combining routine ultrasonography with a TSH-first testing approach—explored according to risk level. Specifically, distinct risk stratifications and screening strategies could be considered based on variations in gender, age, and other risk factors. As a hypothesis for future validation, implementing such differentiated strategies for distinct risk populations may help allocate medical resources more efficiently toward high-risk groups, potentially improving the cost-effectiveness of thyroid screening.

### Research registration unique identifying number (UIN)

UIN: MR-21-26-007262; available at: https://www.medicalresearch.org.cn/.

## Data Availability

The data that support the findings of this study are available on request from the corresponding author. The data are not publicly available due to privacy or ethical restrictions. Requests to access the datasets should be directed to Zhijun Hong, hongzhijun@firsthosp-dmu.com.
